# International initiatives on bridging knowledge, policy and practice

**DOI:** 10.5334/ijic.1084

**Published:** 2012-11-16

**Authors:** Mathilde Leonardi, Jerome Bickenbach, Barbara LeRoy

**Affiliations:** Public Health, Disability Unit, Scientific Director Coma Research Centre, Director Italian WHO-FIC Collaborating Centre Research Branch, Fondazione IRCCS Istituto Neurologico Carlo Besta, Milan, Italy; Disability Policy Unit, Swiss Paraplegic Research, Department of Health Sciences and Health Policy, University of Lucerne and SPF, Nottwil, Switzerland; Developmental Disabilities Institute, Wayne State University, Detroit, Michigan, USA

The principal driver for the growing awareness of the need for international initiatives on bridging knowledge, policy and practices in ageing and disability, and thus the inspiration for the Toronto Declaration [1], is neither political nor professional. It is a matter of hard facts that are not in dispute: the world’s population is ageing, the trajectory of ageing is not consistent across countries, but displays clear social and economic gradients, and ageing is inexorably a matter of acquiring more disability. In 2010, an estimated 524 million people were aged 65 or older, or 8% of the world’s population, and by 2050, this number will triple to 1.5 billion, or 16% of the world’s population [2]. In this time period, the number of older people in low resource countries is expected to increase more than 250%, while in high resource countries it will increase by 71% [2].

The populations of the 48 countries with the least resources are the fastest growing in the world. In China and India combined, by 2050 the population over 65 will account for nearly 25% of the overall world population. The oldest old population is growing the fastest: by mid-century there will be 100 million Chinese over the age of 80, and the global number of centenarians is expected to increase 10-fold [3, 4]. Although life expectancy remains low in low resource countries, increasing longevity globally is a primary cause of this population growth [3]. Given the current best estimate of global disability prevalence at 15–17%, or approximately a billion people worldwide [5], the combination of these numbers is the best argument that could be made for the need to bridge ageing and disability research, practice and policy.

Although each country must face the challenges to its social and health systems of increasing longevity and chronic health conditions, the increased interconnectedness of the world makes these challenges international in scope. It is for this reason that the Toronto Declaration calls for “an international agenda for bridging ageing and disability be formally developed through the involvement of researchers, practice professionals, policy-makers, older adults, persons with disabilities and their families” [1].

There are several on-going international initiatives that create, not merely the context for bridging ageing and disability, but also the potential for worldwide, coordinated and cooperative implementation of integrated ageing and disability policy, practice and research. These initiatives touch on each of the priority areas addressed in the Toronto Declaration: 1) health and well-being, 2) inclusion, participation, and community, 3) long-term supports and services, 4) income security, and 5) the science of bridging.

In December 2010, the United Nations General Assembly established by resolution 65/182 the Open-ended Working Group on Ageing to assess whether the human rights of older persons were adequately addressed in existing international conventions and declarations or whether further measures were required [6]. This initiative was the result of a process, begun in 2002 with the Madrid Plan of Action [7], to bring issues of ageing into the human rights and development domains. At the recent third session of the Working Group in August 2012, they examined age discrimination, autonomy and independent living, dignified lifestyles, social security and access to resources, and abuse and violence [8]. An important spin-off of the Working Group is the Expert Group on the Human Rights of Older Persons jointly convened by the Office of the High Commission for Human Rights (OHCHR) and the UN Department of Economic and Social Affairs (UNDESA). This group is charged with investigating the extent to which the structure of UN human rights instruments deals with age discrimination in general and discrimination in access to health and long-term care facilities for older persons [9].

At both of these UN events, several delegates argued that many of the core rights that were being sought for older people closely reflected the provisions of the UN *Convention on the Rights of Persons with Disabilities* (CRPD) [10]. The case was made for the commonalities between people with disabilities and people who are ageing in terms of the potential need to protect them against similar kinds of discriminatory practice such as demeaning people by denying them dignity, limiting their participation by ignoring their needs for accessibility and accommodation, and engaging in discriminatory practices that drive both populations further and further from the mainstream in employment, community, and political participation [11].

Among the specialized UN agencies, the World Health Organization (WHO) has been the most active in introducing initiatives both in ageing and in disability. In a Discussion Paper on Health and Ageing released in 2002, the WHO introduced into the international policy vocabulary the notion of ‘active ageing’ [12]. Recognizing that ageing as a biological process is one characterized by the increased likelihood that a person will experience minor or major disabilities, the Discussion Paper argued that policies designed to “optimize opportunities for physical, social and mental well-being” were essential [12]. It did not seek to promote the prevention or cure for functional decrements due to ageing, but rather to make possible the best and most ‘active’ life for those entering older age. The resulting Active Ageing Policy Framework (2005) launched initiatives for age friendly primary health care and age friendly cities. Both initiatives recognized the close relationship between moderate and severe impairments and the ageing process, noting that policies promoting accessibility and inclusion are beneficial to both the ageing population and the population of people with disabilities.

A recent joint publication of the WHO and the US National Institute on Ageing, *Global Health and Ageing* [3], presents the best epidemiological evidence available on population ageing trends and the epidemiological case for bridging ageing and disability. Asking hypothetically whether increased longevity has been purchased at the cost of more years spent in poor health, the Report argues for the conceptual need to move beyond vague and idealistic conceptions of health that equate health with overall well-being and to proceed to the more scientifically justifiable understanding of health as grounded in multidimensional human functioning along a continuum. In this conception, ageing is a process of decline of functioning, but what remains unclear is the speed of that decline. As people live longer, does functional decline accelerate or does it tend to plateau until the very last period of life when disability is ‘compressed’ into a very quick decline [13]?

While the idea of disability ‘compressing’ has profound and far reaching consequences for social policy, what is more significant is the terms in which it is expressed: ageing, though inevitably a process of health decline, is not always a matter of precipitous decline. What this means is that people will not only live longer, they will live longer with impairments that range from the mild joint pains and decline in visual and hearing acuity, to moderate and severe impairments.

The final, and in many respects more significant contribution in the effort to bridge ageing and disability is WHO’s conception of functioning and disability in its International Classification of Functioning, Disability and Health (ICF) [14]. The ICF’s primary contribution to practice and research on disability is as an international classification that makes it possible to compare data on health and disease globally and to standardize the ‘language of disability’. The ICF, therefore, is the international solution to the challenge expressed in the Toronto Declaration of having a ‘common terminology and knowledge base’ that can be used to bridge ageing and disability [1]. More importantly still, the conceptual model in the ICF with its reliance on the central notion of continuous and multidimensional human functioning not only provides the only scientifically legitimate concept of disability, but it is also the basis by which ageing and disability can be conceptually linked.

Further, the WHO has also recently contributed to the knowledge translation literature focusing on the ageing and disability bridging issue [15]. Knowledge translation is not intended as a way of bridging ageing and disability directly, but rather is a means for assisting policy- and decision-makers in integrating ageing and disability research into national policies or specific programs targeting older populations or persons with disabilities [16]. As such, WHO’s *Translation on Ageing and Health: a framework for policy development* is a valuable contribution to the bridging effort.

Another important document addressing ageing and disability was developed by the Organization for Economic Cooperation and Development (OECD). In this study, they examined the trends among the 12 OECD countries between severe disability among elderly people and the projected social costs of health and long-term care and ageing related social programs [17]. For a careful, analytic breakdown of the ramifications of ageing on pensions, healthcare and long-term care—and so an integration of knowledge between ageing and disability—the most reliable international work has been done by the Geneva Association for Risk and Insurance Research [18].

Finally, in Europe there are several very significant initiatives that are consistent with the aspirations and objectives of the Toronto Declaration. The European Union has declared 2012 as the European Year for Active Ageing and Solidarity between Generations (EY2012), which aims to establish a framework for bridging ageing and health and employment issues, raising awareness, identifying and disseminating good practice, and encouraging policy-makers and stakeholders at all levels to promote active ageing and solidarity between generations [19]. The European Innovation Partnership on Active and Healthy Ageing [20], part of the European Commissions ‘Innovation 2020’ long range research agenda, seeks to link researchers, practitioners and policy-makers across Europe in both ageing and health fields to encourage cooperation based on a shared vision and common targets, while fostering synergies and avoiding overlaps.

These highlights of international initiatives that seek to facilitate, secure and maintain connections between the research, practice and policy communities from ageing and disability demonstrate the relevancy of the recommendations of the Toronto Declaration. Each, in different ways and subject to different political and cultural pressures, seeks what the Toronto Declaration aspires to achieve: a conceptual and practical common ground, in which ageing and its stakeholders and champions can join forces with those of disability to secure not only the efficiencies of common cause and integrated research and practice, but ultimately the improvement of the lives of all of us as we age and as we acquire those functional limitations that define our conditions as human beings.
